# Expression of immunogenic structural proteins of cyprinid herpesvirus 3 in vitro assessed using immunofluorescence

**DOI:** 10.1186/s13567-015-0297-6

**Published:** 2016-01-08

**Authors:** Sean J. Monaghan, Kim D. Thompson, James E. Bron, Sven M. Bergmann, Tae S. Jung, Takashi Aoki, K. Fiona Muir, Malte Dauber, Sven Reiche, Diana Chee, Shin M. Chong, Jing Chen, Alexandra Adams

**Affiliations:** Institute of Aquaculture, School of Natural Sciences, University of Stirling, Stirling, FK9 4LA UK; Moredun Research Institute, Pentlands Science Park, Bush Loan, Penicuik, EH26 0PZ UK; Friedrich-Loeffler-Institut, Federal Research Institute for Animal Health, Institute of Infectology, Greifswald, Insel-Riems Germany; Laboratory of Aquatic Animal Diseases, Institute of Animal Science, College of Veterinary Medicine, Gyeongsang National University, Jinju, Gyeongnam South Korea; Consolidated Research Institute for Advanced Science and Medical Care, Waseda University, 513, Wasedatsurumaki-cho, Shinjuku-ku, Tokyo, 162-0041 Japan; Aquatic Animal Health Section, Animal Health Laboratory Department, Laboratories Group, Agri-Food and Veterinary Authority of Singapore, Singapore, Singapore; Virology Section, Animal Health Laboratory Department, Laboratories Group, Agri-Food and Veterinary Authority of Singapore, Singapore, Singapore

## Abstract

Cyprinid herpesvirus 3 (CyHV-3), also called koi herpesvirus (KHV), is the aetiological agent of a fatal disease in carp and koi (*Cyprinus carpio* L.), referred to as koi herpesvirus disease. The virus contains at least 40 structural proteins, of which few have been characterised with respect to their immunogenicity. Indirect immunofluorescence assays (IFAs) using two epitope-specific monoclonal antibodies (MAbs) were used to examine the expression kinetics of two potentially immunogenic and diagnostically relevant viral antigens, an envelope glycoprotein and a capsid-associated protein. The rate of expression of these antigens was determined following a time-course of infection in two CyHV-3 susceptible cell lines. The results were quantified using an IFA, performed in microtitre plates, and image analysis was used to analyse confocal micrographs, enabling measurement of differential virus-associated fluorescence and nucleus-associated fluorescence from stacks of captured scans. An 8-tenfold increase in capsid-associated protein expression was observed during the first 5 days post-infection compared to a ≤2-fold increase in glycoprotein expression. A dominant protein of ~100 kDa reacted with the capsid-associated MAb (20F10) in western blot analysis. This band was also recognised by sera obtained from carp infected with CyHV-3, indicating that this capsid-associated protein is produced in abundance during infection in vitro and is immunogenic to carp. Mass spectrometry carried out on this protein identified it as a previously uncharacterised product of open reading frame 84. This abundantly expressed and immunogenic capsid-associated antigen may be a useful candidate for KHV serological diagnostics.

**Electronic supplementary material:**

The online version of this article (doi:10.1186/s13567-015-0297-6) contains supplementary material, which is available to authorized users.

## Introduction

Cyprinid herpesvirus 3 (CyHV-3) is the taxonomical classification for koi herpesvirus (KHV) [1], the aetiological agent of an economically important and often fatal disease, koi herpesvirus disease (KHVD), in common carp and koi (*Cyprinus carpio* L.) worldwide [2–4] and is a member of the *Alloherpesviridae* family of the order *Herpesvirales* [1, 5].

Analysis of herpesviruses during the infectious cycle can provide an insight into the role of the various proteins of the virus and indicate which stages of infection they are important to with regards to virulence and antigenicity. Virions of herpesviruses have large genomes, generally encompassing around 40 genes that encode for structural proteins. Some of these proteins execute similar functions in the replication cycle between the various families, but recent applications of mass spectrometry have identified differences in the proteins that make up the capsid, tegument and envelope of the virion of different herpesviruses [6–10]. The proteome of CyHV-3 has been shown to consist of 2 tegument, 3 capsid and 13 envelope proteins [8], although more recent analysis identified 16 envelope proteins in a Chinese isolate [10]. The role of the 22 other proteins have yet to be determined and the antigenic characteristics and biological function of many of the proteins, with regards to the virus replication, have not yet been elucidated. Molecular analysis of the genes expressed by CyHV-3 has enabled the open reading frames (ORFs) of the virus to be characterised [11, 12]. Such analysis does not, however, take into account post-transcriptional processing such as translation initiation, elongation and termination [13], and up to 60% of the variation in protein concentration cannot be explained from mRNA analysis alone [14]. Thus, the use of monoclonal antibodies (MAbs) may provide a useful/supplementary source of data with respect to the expression characteristics of some of these ORFs. It has already been shown that some CyHV-3 expression profiles, differ at the protein level [10] with respect to the transcript level [11], using rabbit anti-sera against specific CyHV-3 proteins in western blot analysis.

There still remains a great deal to be elucidated with respect to the immunogenicity of CyHV-3 proteins. Although the product of ORF81 is thought to be an immunodominant protein [15], recent studies have revealed a number of envelope glycoproteins recognised by infected carp anti-sera, encoded by CyHV-3 ORFs-25, -65, -148 and -149 and the major capsid protein encoded by ORF92 [16]. In addition, a non-structural protein, encoded by ORF12, has also been recognised by serum from infected carp [17]. Determination of which immunogenic antigens are expressed in highest abundance during the lytic infectious cycle may provide useful targets for the development of serological diagnostic tests.

Previous reports on the use of MAbs in CyHV-3 research have tended to focus on the development of diagnostic tests [18–20], diagnosis [21], major glycoprotein and capsid characterisation [15, 22], protein affinity purification [23] and screening of recombinant mutants [24]. Relatively few studies have used MAbs to investigate aspects of CyHV-3 biology, replication and maturation [10, 16]. Thus, two MAbs were employed in the current study to assess the expression kinetics of two potentially diagnostic-relevant CyHV-3 antigens. These proteins, having contrasting roles in virion assembly, were examined during the course of the CyHV-3 lytic infectious cycle in vitro, to determine their expression pattern, identity and potential immunogenicity in carp.

## Materials and methods

### Cell culture

Koi fin cells (KF-1 cells), developed from epidermal tissue of koi [2] were kindly provided by Dr Keith Way [Centre for Environment, Fisheries and Aquaculture Science (CEFAS), Weymouth, UK]. Common carp brain cells (CCB cells) developed from brain tissue of Common carp, *Cyprinus carpio* L. [25] were kindly provided by Dr Matthias Lenk (Friedrich Loeffler Institut, Greifswald, Germany). Cells were cultured in Eagle’s Minimum Essential Medium (EMEM) containing Eagles’s salts (Invitrogen), 10% foetal bovine serum (FBS), 1% non-essential amino acids (NEAA, Invitrogen) and 2 mM l-glutamine at 22–25 °C with 4% CO_2_. CCB cells were maintained between a passage of 108–144, while KF-1 cells were maintained between a passage of 69 and 84.

### Virus culture and purification

An American isolate of CyHV-3 (KHV, H361), originating from an adult koi with KHVD in Eastern USA in 1998 [2], was used in the study. Cells were maintained at 22 °C, then culture medium was removed and monolayers washed with Dulbecco’s phosphate buffered saline (DPBS) prior to inoculation with the virus. An adsorption period of 1–2 h at 20 °C was performed before re-supplementing infected cultures with fresh EMEM, which contained 2% FBS. The virus was harvested when a cytopathic effect (CPE) of 90–100%, consistent with CyHV-3, was obtained. The infected cultures were exposed to two freeze/thaw cycles at −70 °C, and then centrifuged at 3800 ×* g* (Eppendorf 5804 R). The clarified supernatants and pellets were collected and stored at −70 °C.

For virus quantitation, KF-1 and CCB cells were cultured overnight at 22 °C in 24 or 96 well tissue culture plates (Nunc, Denmark). The cells were inoculated with a fivefold serial dilution of clarified virus supernatant as described above with control cells receiving only diluent. The tissue culture infectious dose (virus infection of 50% inoculated cells (TCID_50_)) was determined after 7 and 14 dpi according to Spearman-Kärber [26]. Multiplicity of infection (MOI) was determined as described by Voronin et al. [27].

The pellets from harvested virus were dissolved in 1 mL TNE buffer (10 mM Tris, 10 mM NaCl, 3 mM EDTA, pH 7.4) and sonicated to further disrupt the lysed cells using 4 × 30 s pulses in an ultrasonicator bath (Kerry, Guyson, Yorkshire, UK) filled with iced water. The cell suspension was centrifuged at 2000 × *g* for 10 min at 10 °C and the pellets discarded. This supernatant was combined with the original clarified virus supernatant and was centrifuged in an ultracentrifuge at 12000 × *g* for 35 min at 4 °C in a SW28 rotor (Beckman L-80). The supernatant was then centrifuged on a SW41Ti rotor at 100000 × *g* for 50 min at 4 °C (Beckman Coulter). The pellets were retained, air dried for 5 min and dissolved in 0.5 mL TN buffer (10 mM Tris, 10 mM NaCl, pH7.4).

The virus suspension was layered onto 20–60% discontinuous sucrose gradients in TN buffer and centrifuged at 110000 ×* g* for 1 h at 4 °C on a SW41Ti rotor. Bands containing CyHV-3 virions, located at around 40 and 50%, were collected by puncturing the tube with a 16G needle (Terumo) and diluted with fresh TN buffer. The CyHV-3 suspension was centrifuged at 100000 × *g* for 60 min at 4 °C and the supernatant discarded. The resulting pellets were dissolved in 1 mL TN buffer and the purified virus quantified with a Pierce BCA protein assay kit (Thermo Scientific, Rockford, USA) using bovine serum albumin (BSA) as a standard according to the manufacturer’s instructions. Total yields of purified CyHV-3 protein ranged between 0.9 and 1.4 mg mL^−1^. The purified virus was stored at −70 °C.

### Monoclonal antibody (MAb) production and screening

Hybridoma cells producing MAbs to CyHV-3 capsid-associated protein (MAb 20F10) and envelope glycoprotein (MAb 10A9) antigens [16], were produced at the Friedrich Loeffler Institut, Greifswald, Germany [16]. The hybridoma’s were cultured at 37 °C in 5% CO_2_ in Dulbecco’s Minimum Essential Medium plus additive (DMEM+) (Sigma-Aldrich, St. Louis, USA), containing 10% (v/v) foetal calf serum (FCS) (Sigma-Aldrich), 2.5 mL penicillin streptomycin (PenStrep) 1250 units (U) (10000 U penicillin; 10 mg mL^−1^ streptomycin (Sigma-Aldrich)), 5 mL l-glutamine (200 mM) (Sigma-Aldrich) and 5 mL sodium pyruvate (100 mM) (Sigma-Aldrich).

The MAbs were affinity purified using 1 mL affinity purification High Trap Protein-G columns (GE Healthcare) on an ÄKTA prime liquid affinity chromatography system (Amersham Biosciences). The IgG bound to the column was eluted as 1 mL fractions using Glycine–HCl, pH 2.7. The eluted fractions were neutralised with 100 µL Tris buffer, pH 9. Fractions containing purified MAbs, determined from their absorbance at OD_280_, were pooled and dialysed against phosphate buffered saline (PBS) 0.02 M phosphate, 0.15 M NaCl, pH 7.2. The concentration was then determined using a Pierce BCA protein assay kit.

### Microtitre plate assay

#### Preparation of infected cells

The CCB and KF-1 cells were seeded at 2 × 10^4^ and 1.5 × 10^4^ cells well^−1^ for KF-1 cells and CCB cells, respectively. Both cell lines were inoculated with CyHV-3 at an MOI of approximately 0.1 using HBSS, 2% FBS as diluent with controls receiving diluent only.

Black immunofluorescence 96-well tissue culture plates (Greinar Cellstar^®^) were used for analysing CyHV-3 antigens expressed from 0 to 24 h post infection (hpi) and 2–7 dpi. An additional plate of cells was mock-infected to analyse non-specific binding of MAbs.

Before initiating the time trial, medium was removed from the first row of wells and the cells were washed twice with DPBS then fixed with cold (−20 °C) methanol. These cells were designated as time point “0 hpi”. Medium was then removed from all remaining wells on the plate and 100 µL of CyHV-3 was added to the monolayers at an MOI of 0.1. The mock-infected control wells received only HBSS, 2% FBS. The time trial was initiated from this point forward, with cells incubated at 20 °C. After 1 hpi the medium was removed from one row of the infected cells, washed twice with DPBS and fixed with cold methanol and the plate returned to 20 °C. The procedure was repeated for another row of wells at 2 hpi. Cells were washed twice with DPBS and culture medium containing 2% FBS was re-supplemented to all other cells after 2 hpi, incubating plates at 20 °C. Subsequent rows were treated in a similar manner as above at 4, 6, 8 and 24 hpi, respectively with one 96-well plate, and then at 2, 3, 4, 5, 6 and 7 dpi, respectively with the second 96-well plate. The final row on each set of plates was fixed after 10 dpi to ensure that antigen detection by MAbs was similar for both sets of plates. Fixed cells in plates were stored at −20 °C till analysis.

#### IFAT on fixed cells

Plates were thawed, rehydrated and washed 4 × 2 min with 300 µL well^−1^ PBST (0.01 M PBS, 0.05% Tween-20). The cells were treated with 250 µL well^−1^ 5% skimmed milk powder (SMP) in PBST for 1 h at 22 °C. After a further wash with PBST the two affinity-purified MAbs (20 µg mL^−1^) were added to the plate (100 μL) and incubated for 1 h at 22 °C. The plates were washed again and 100 µL goat anti-mouse IgG fluorescein isothiocyanate (FITC)-conjugated MAbs (Sigma-Aldrich, USA), diluted 1/100 in PBS, were added to the wells and incubated for 1 h at 22 °C. The plates were washed a final time and kept in the dark at 4 °C prior to reading the fluorescence in a Synergy HT spectrophotometer (Fisher Scientific, Leicestershire, UK). The Gen 51.10 program was used for data acquisition at a fluorescence detection sensitivity setting of 120, with filter settings applied at wavelengths of 485/20 excitation and 528/20 emission.

Once the FITC fluorescence had been measured, 50 µL DAPI mountant (Vectashield, Vector, UK), diluted 1:10 in PBS, was added to all wells and incubated at 22 °C for 10 min. Excess DAPI was removed from the wells by 4 × 2 min washes with PBST. The plates were read a second time using the same program and sensitivity settings, but with filter sets of 360/40 excitation and 460/40 emission.

#### FITC:DAPI quantification for specific CyHV-3 antigen detection

The formula below utilised by Xijier et al. [28] for quantifying structural proteins of Rotavirus (RV) by fluorescent labelling with MAbs, was applied:

$$^{\text{V}} {\text{FITC}}/{\text{DAPI}} - ^{\text{B}} {\text{FITC}}/{\text{DAPI}}.$$

where V = measurement ratio of the presence of virus and B = blank control (0 hpi fixation).

### Confocal microscope assay

#### Infection of cells on cover slips

Transparent 12-well plates (Nunc, Denmark) were used to culture KF-1 cells and CCB cells seeded onto sterile 1.6 mm^2^ glass cover slips (Fisher Scientific, UK) for 24 h at 20 °C. The KF-1 cells were seeded with 3.6 × 10^5^ cells well^−1^ and CCB cells with 1.4 × 10^5^ cells well^−1^.

Culture medium was removed from the wells and 6 of the 12 wells were inoculated with 0.2 mL CyHV-3 at an MOI of 0.01–0.02, while the other six were inoculated with diluent only (HBSS, 2% FBS) to serve as mock infected controls. The cells were incubated at 20 °C for 2 h for viral adsorption to the monolayer. After 1 h, the inoculum was removed from 1 plate of each cell line, cells were washed twice with 1 mL DPBS and fixed with 500 µL 100% cold acetone (−20 °C) (Fisher Scientific, UK). The cells were fixed for 15 min at 22 °C before air drying for 30 min at 22 °C. For the other plates, cells received 2× washes with DPBS then 1.8 mL of EMEM media containing 2% FBS and the plates were incubated at 20 °C with 4% CO_2_. The fixation procedure was repeated randomly for these plates at time points of 4 and 8 hpi then 1, 3, 5 and 7 dpi. All fixed plates were stored at −20 °C until processing.

#### IFAT and confocal microscopy

Fixed cells were rehydrated for 5 min with 1 mL PBST. The cells on cover slips were washed 3× PBST for 2 min and were covered with 1 mL 5% SMP diluted in PBST and incubated for 30 min at 37 °C. Cells were washed again before adding 250 µL of purified MAbs at 20 µg mL^−1^. The MAb preparations were added to both CyHV-3 positive and mock infected cells to determine the specificity of signal. Additionally, a MAb with the same isotype, detecting a different virus (i.e. infectious salmon anaemia virus (Aquatic Diagnostics Ltd., Stirling, Scotland)) and PBS were added as negative controls. After 1 h incubation at 22 °C, the cells were washed and 1 mL 1/100 goat anti-mouse IgG conjugated to FITC (Sigma-Aldrich, USA) was added and cells were incubated for 1 h at 22 °C. The cells were washed a final time and mounted onto slides (Solmedia, Shrewsbury, UK) in 20 µg mL^−1^ propidium iodide (Sigma-Aldrich, UK) diluted in PBS and sealed with nail varnish (Avon, UK). All slides were kept in the dark at 4 °C until visualised. Analysis was carried out using a confocal microscope (Leica TCS SP2 AOBS confocal laser scanning microscope (CLSM, Germany)) coupled to a DM TRE2 inverted microscope (Leica Microsystems, Milton Keynes, UK) and employing a X 63 oil/glycerol immersion objective, in conjunction with Leica confocal software (v. 621).

Confocal microscopy and image analysis were performed according to methods described previously [29]. Images were captured in the grey (transmission), red, green and blue channels using the relevant excitation and emission wavelengths for the respective dye, depending on the target. A sequential scanning configuration was used with images collected successively rather than simultaneously on three separate channels. At least two replicate images per culture well were captured, including positive, negative and control cells from each cell line at each time point post-infection. Replicate experimental cultures were taken for the KF-1 cell line, but not the CCB cell line. Stacks of 25 serial depth images (*z*-stacks) were taken from each sample of cells by scanning a frame area of 1024 × 1024 pixels (*x* × *y* μm) in the *x*, *y* plane. The stacks of images through the cells had a total depth of 25 µm comprising 25 transects of 1 µm moving from the basal surface of the cell to the apical surface. Prior to image analysis, the grey channel from each image was removed and the stacks were collapsed to give a projection of maximal fluorescence intensity for the stack as a single 2-D image (Leica Maximum Projection).

#### Image analysis of confocal micrographs

The Carl Zeiss KS 300 image analysis platform was used for image analysis, employing a custom macro script, which enabled quantification of a number of morphometric and densitometric features of the target fields (e.g. whole image) or individual objects (e.g. nuclei).

For quantifying nuclear signal intensity, the nuclei were segmented from the background using a HLS colour segmentation function and the adjoining nuclei were separated from one another using a grain separation function and subjected to size thresholding in order to exclude noise. The final segmented areas were used as a field for densitometric measurements of nuclei and nuclear fragments enabling measurements of mean total nuclear fluorescence per section. The separation function was used in a similar manner for isolating MAb-associated signals in order to measure the level of virus antigens through use of MAb signal intensity as a proxy.

Quantification of fluorescence signals associated with nuclear staining (propidium iodide) and antibody-antigen complexes of CyHV-3 (FITC) was achieved using the macro described above to provide five parameters measured from the replicate scans (*n* = 2 per slide). Data from three of these parameters:NUC (the total nuclear area), MBD (the average intensity of the total cell fluorescence, including cytoplasm, exhibited by the MAb) and MND (the average intensity of nucleus-only fluorescence exhibited by the MAb) proved useful for quantification of virus-associated signal by allowing determination of differences in relative MAb fluorescence (MND or MBD) compared to nuclear fluorescence (NUC) (representing cell confluence) and finding the difference between infected samples and negative controls using the following formula:

$$^{\text{V}} {\text{MND}}/{\text{NUC}}{-}^{\text{C}} {\text{MND}}/{\text{NUC or}}^{\text{V}} {\text{MBD}}/{\text{NUC}}{-}^{\text{C}} {\text{MBD}}/{\text{NUC}}.$$

where V = measurement ratio of the presence of virus in infected cells and C = measurement ratio of the presence of autofluorescence signals/noise in uninfected cells.

### Statistical analysis

Non-parametric data obtained during the trials (relative expression) were analysed using a Kruskal–Wallis test to determine significant differences by analysis of medians of FITC:DAPI and MND/MBD:NUC ratios (relative antigen expression; RAE) during early infection (1–24 hpi) and late infection (2–7 dpi) for MAbs 10A9 and 20F10 (Minitab 16).

### Koi sera

Positive koi anti-CyHV-3 sera and sera from individual naïve koi were obtained from an experimental CyHV-3 challenge carried out in Singapore by the Animal Health Laboratory, the Agri-Food and Veterinary Authority of Singapore (Institutional Animal Care and Use Committee application AVA-APHC-2011-01). These fish had previously been screened for CyHV-3 by real-time TaqMan polymerase chain reaction (PCR) assay [30] and were from a stock of fish with no known exposure to KHV or record of KHVD. The challenge consisted of an infectious dose of 1.2 TCID_50_ mL^−1^ of KF-1 cell propagated CyHV-3 according to the method of Reed and Muench [31]. Blood was sampled from the caudal vein of fish after 16 dpi, allowed to clot overnight at 4 °C, and serum collected by centrifuging (Microlite, Thermo IEC, USA) blood at 850 × *g* for 5 min. Sera were stored at −70 °C until analysis. The sera from 1 CyHV-3 infected and naïve koi were used for the western blot analysis.

### Sodium dodecyl sulphate–polyacrylamide gel electrophoresis (SDS-PAGE)

One hundred microliters of both purified CyHV-3 (0.5 mg mL^−1^) and lysed suspensions of 4 day old CCB cells were combined with 100 µL of 2× SDS sample buffer (0.5 M Tris–HCl, pH 6.8, 20% glycerol, 4% SDS, 0.2 M dithiothreitol, 0.02% bromophenol blue). Reduced proteins were boiled for 2 min and then centrifuged (Microlite, Thermo IEC, US) for 5 min at 16000 ×* g*.

SDS-PAGE, was performed according to Laemmli [32], with two gels prepared using Pro-Pure Next Gel™ 12.5% kit (Amresco, USA) and run at 175 V for 75 min using a Hoefer SE250 mini-vertical gel electrophoresis unit (Hoefer, USA) according to the manufacturer’s instructions. Precision Plus Protein™ Kaleidoscope™ Standards (BioRad, USA) were included in each gel. Gels were stained with silver using the ProteoSilver™ silver stain kit (Sigma-Aldrich, USA) according to the manufacturer’s instructions.

### Western blot

Western blot was carried out using CyHV-3 capsid-associated protein MAb 20F10 and envelope glycoprotein specific MAb 10A9 or koi sera. Purified CyHV-3 and CCB cell lysate were used as antigen. Polyacrylamide gels containing separated proteins (section “Sodium dodecyl sulphate–polyacrylamide gel electrophoresis (SDS-PAGE)”) were transferred to nitro-cellulose membranes (Amersham™ Hybond™ ecl, GE Healthcare, UK) by applying 60 V for 30 min in transblot buffer (25 mM Tris, 192 mM glycine, 20% v/v methanol, pH 8.3) using a wet blotting apparatus (Fisher Brand, UK) according to the manufacturer’s instructions. After blotting, the membranes were blocked overnight at 4 °C with 2% SMP for MAb screening, or 5% SMP for sera screening, in Tris buffered saline (TBS, 0.02 M Trisma base, 0.5 M NaCl, pH 7.5). The following day the membranes were washed 3× with TBST (TBS containing 0.1% Tween-20) for 5 min and cut into strips, which were incubated with either 20 µg mL^−1^ MAbs in TBS for 1 h at 22 °C or sera (1/50 or 1/200 in either TBST or 2% SMP in TBST) for 3 h at 22 °C. For sera screening, another wash was performed and fish antibody detected using an anti-carp IgM MAb (ADL, Stirling, Scotland) diluted 1/50 in TBST for 1 h at 22 °C. Control strips were incubated with PBS (background controls).

The membranes were washed again then incubated for 1 h at 22 °C with goat anti-mouse IgG biotin (Sigma-Aldrich, UK) diluted 1/200 in TBS. After another wash, the membranes were incubated with streptavidin-horseradish peroxidise (Streptavidin-HRP, Vector Labs, USA) diluted 1/200 in TBS for 1 h at 22 °C. The membranes were washed a final time including a 1 min wash without tween-20 and developed using the 4 CN peroxidase substrate system (2-C: KPL, USA) according to the manufacturer’s instructions. The reaction was stopped after 5–10 min with ultrapure H_2_O.

### MALDI-TOF/TOF mass spectrometry

Selected protein bands, recognised by the infected fish in western blotting, were excised from SDS-PAGE gels stained with Bio-Safe Coomassie G-250 stain (Bio-Rad, USA) and in gel digestion performed with trypsin as previously described [33]. MALDI TOF/TOF MS analysis was carried out as described previously for ranavirus-1 antigens [34].

## Results

### Identification of purified CyHV-3 proteins from SDS-PAGE and silver staining

**Figure 1:**
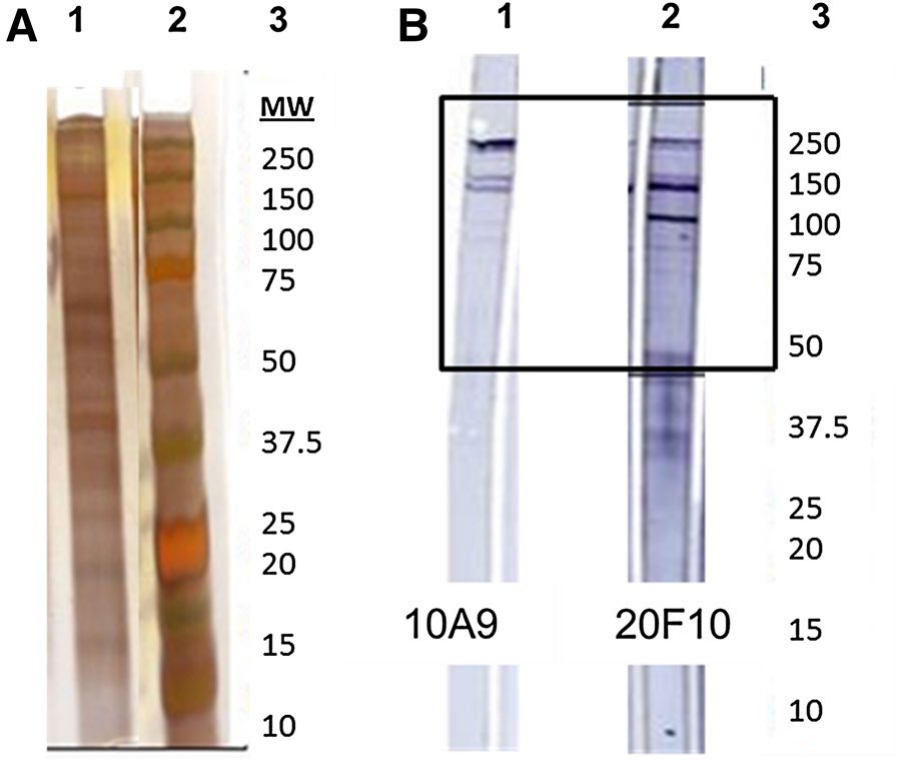
**SDS-PAGE and western blot analysis of sucrose gradient purified CyHV-3 using monoclonal antibodies 10A9 and 20F10.** **A** Silver stained SDS-PAGE of sucrose gradient purified CyHV-3, American isolate H361. Lane 1 CyHV-3 proteins from 0.25 mg mL^−1^ purified CyHV-3; Lane 2 10–250 kDa molecular weight markers; Lane 3 size of protein marker of interest. **B** Western blot analysis of purified MAbs 10A9 and 20F10 screened against purified CyHV-3. Lane 1 MAb 10A9, Lane 2 MAb 20F10, Lane 3 relative size (MW) of proteins on membrane.

Around 20–22 bands were detected in the purified CyHV-3 preparation with SDS-PAGE (Figure 1A), the majority of which were associated with the virus and not the uninfected cells (data not shown).

### Western blot analysis of MAbs against purified CyHV-3 proteins

Both MAb 10A9 and 20F10 recognised bands of 250, 240, 150 and 130 kDa. However, a dominant band of ~100 kDa was only identified by MAb 20F10 (Figure 1B).

### Localisation of antigens on CyHV-3 infected cells

**Figure 2:**
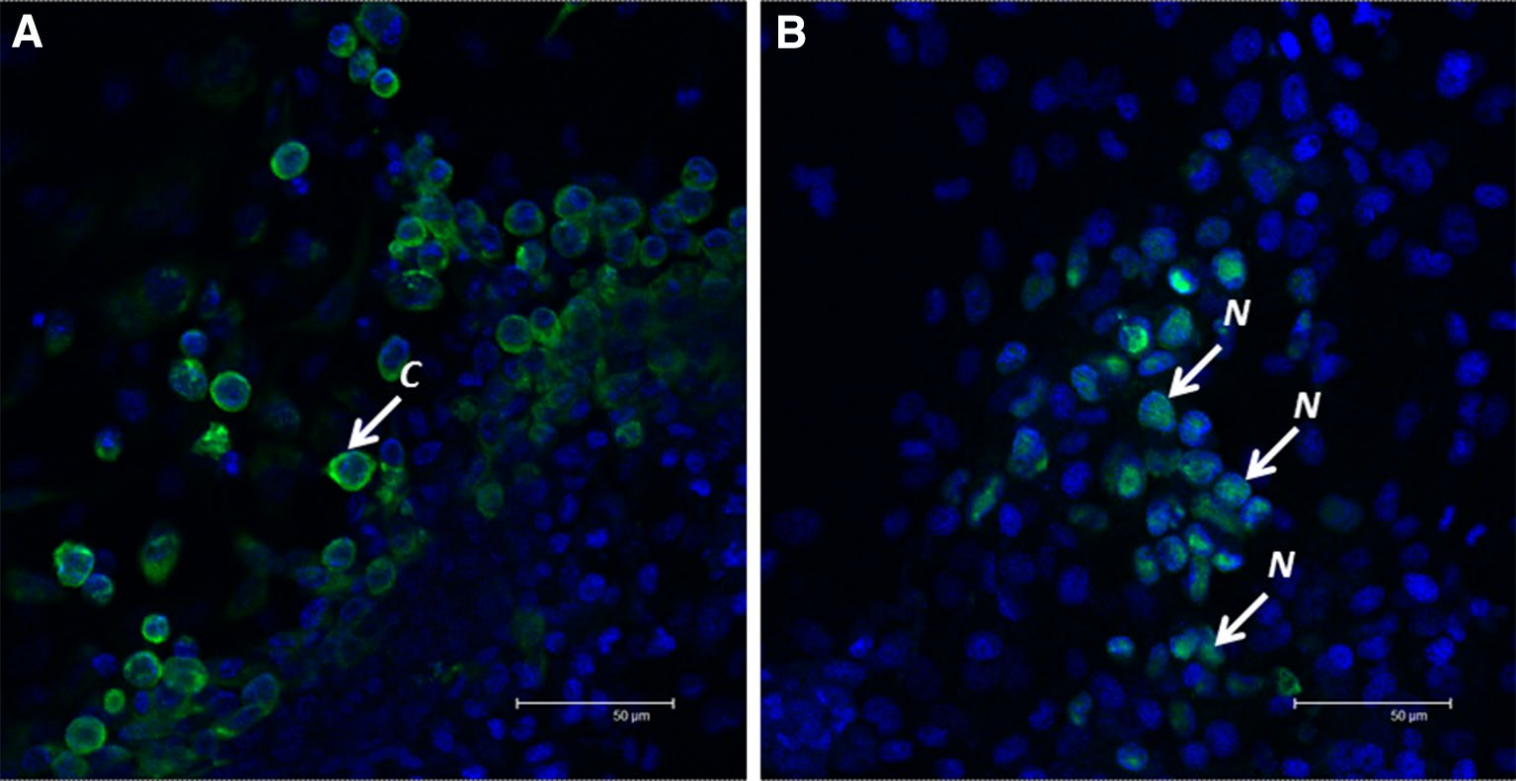
**Confocal micrographs of CyHV-3 infected KF-1 cells (10 dpi) screened by two different monoclonal antibodies.** **A** MAb 10A9, **B** MAb 20F10. Images captured at ×3 zoom. Blue DAPI (nuclei), green FITC (CyHV-3), N nuclear staining, C cytoplasmic staining. Both micrographs show an overlay of green and blue channels.

The antigen recognised by MAb 10A9 was localised in the cytoplasm of cells, often associated with the inner cell membrane (Figure 2A), whereas cytoplasmic and also specific intra-nuclear staining, was observed with MAb 20F10 (Figure 2B) in cells infected with CyHV-3 at 10 dpi.

### Expression of glycoprotein and capsid-associated antigens determined by immunofluorescence in 96-well tissue culture plates

**Figure 3:**
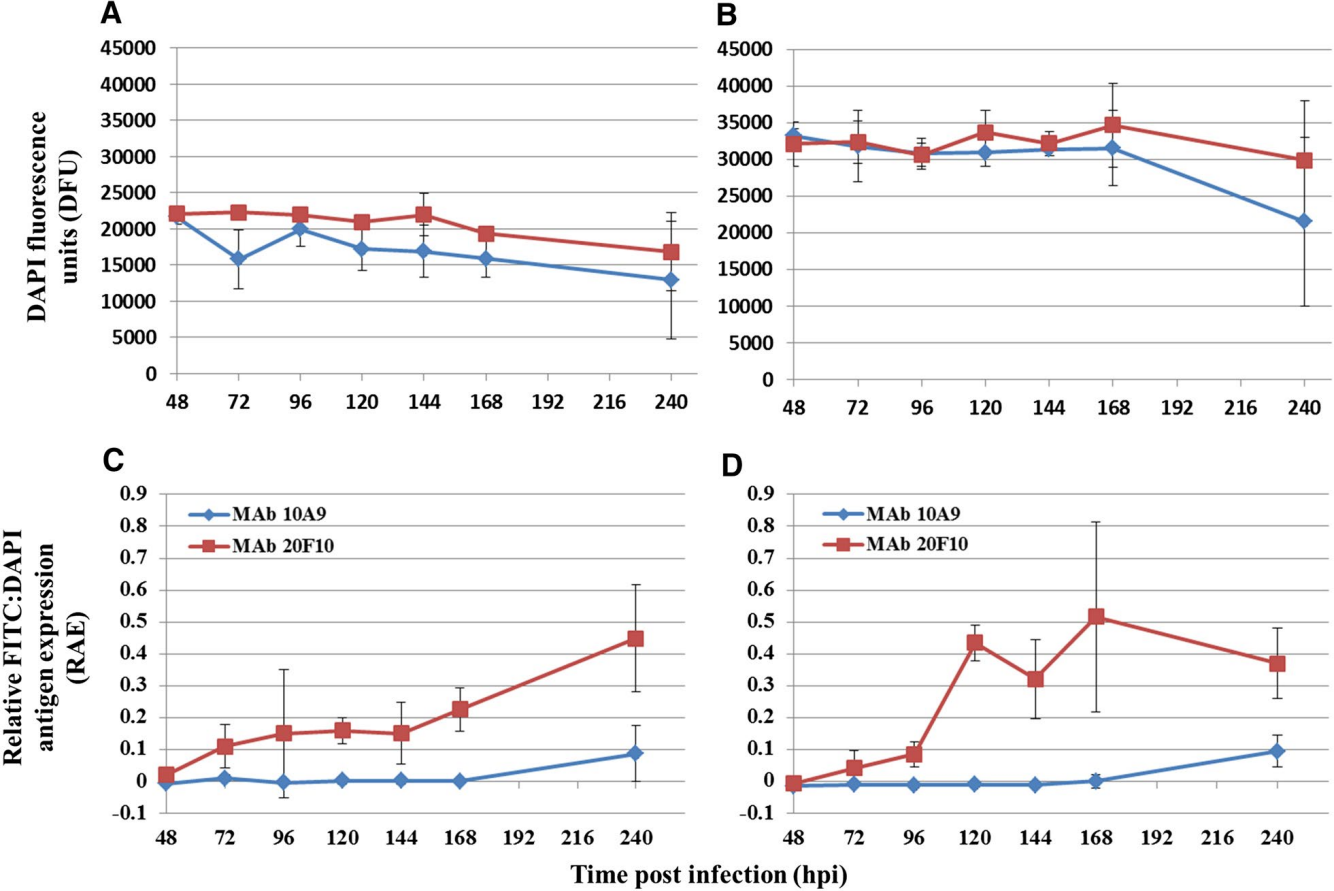
**CyHV-3 antigen expression using immunofluorescence of capsid-associated protein and envelope glycoprotein MAb binding in 96 well microtitre plates.** **A** DAPI fluorescence of KF-1 cells; **B** DAPI fluorescence of CCB cells; **C** difference of relative FITC to DAPI stain from blank wells at 0 hpi in KF-1 cells; **D** difference of relative FITC to DAPI stain from blank wells at 0 hpi in CCB cells. MAb 10A9 recognition of envelope glycoprotein; MAb 20F10 recognition of capsid associated protein. Mean ± SE (n = 4 individual cell cultures).

DAPI fluorescence was relatively stable within the samples during the first day of infection with a gradual reduction in cell nuclear fluorescence from 2–10 dpi (Figure 3A, B) although this was not significantly different from the first day (KF-1 cells, *p* = 0.326; CCB cells, *p* = 0.439).

Increased binding to capsid-associated antigen was detectable as early as 6 hpi by MAb 20F10. In contrast, only minimal binding of MAb 10A9 to envelope glycoprotein antigen was observed during the first 24 hpi. There were no significant differences between glycoprotein and capsid-associated antigen expression during these early stages of infection (KF-1 cells, *p* = 0.141; CCB cells, *p* = 0.082) (see Additional files 1, and 2 for graphs of antigen expression and DAPI fluorescence during the first dpi).

There was a significant increase in capsid-associated antigen expression, detected with MAb 20F10 between 2 and 10 dpi (KF-1 cells, *p* < 0.001; CCB cells, *p* < 0.001) (Figure 3C, D) compared to earlier time-points (i.e. 1–24 hpi) whereas only minimal envelope glycoprotein antigen expression was detected with MAb 10A9 (Figure 3C, D). Significantly greater expression of the glycoprotein antigen was, however, observed at later stages of the infection (i.e. 2–7 dpi) (KF-1 cells, *p* = 0.05), compared to earlier stages (i.e. 1–24 hpi). Notably elevated capsid-associated antigen expression occurred between 4 and 7 dpi (Figure 3C, D) as detected by MAb 20F10.

There was no detectable increase in envelope glycoprotein antigen expression between 1 and 7 dpi, as shown from the binding of MAb 10A9, within the detection limits of the assay. This suggests only minimal abundance of this glycoprotein was apparent until the final sampling point at 10 dpi where an increase in expression of the glycoprotein did occur in conjunction with complete infection of the cell monolayer (Figure 3C, D). Capsid-associated antigen expression was significantly greater than glycoprotein expression during the later stages of infection (i.e. 2–7 dpi) (KF-1 cells, *p* < 0.001; CCB cells, *p* < 0.001).

### Expression of glycoprotein and capsid-associated antigens determined by confocal microscopy and image analysis

Data from different parameters, NUC, MND and MBD (explained in section “Image analysis of confocal micrographs”), were obtained from confocal micrographs after transforming fluorescence from propidium iodide and FITC into values, which were then used in the image analysis. An additional figure shows the transformation of micrographs displaying fluorescence emitted from nuclei and virus antigens into black and white images compatible for image analysis (Additional file 3).

**Figure 4:**
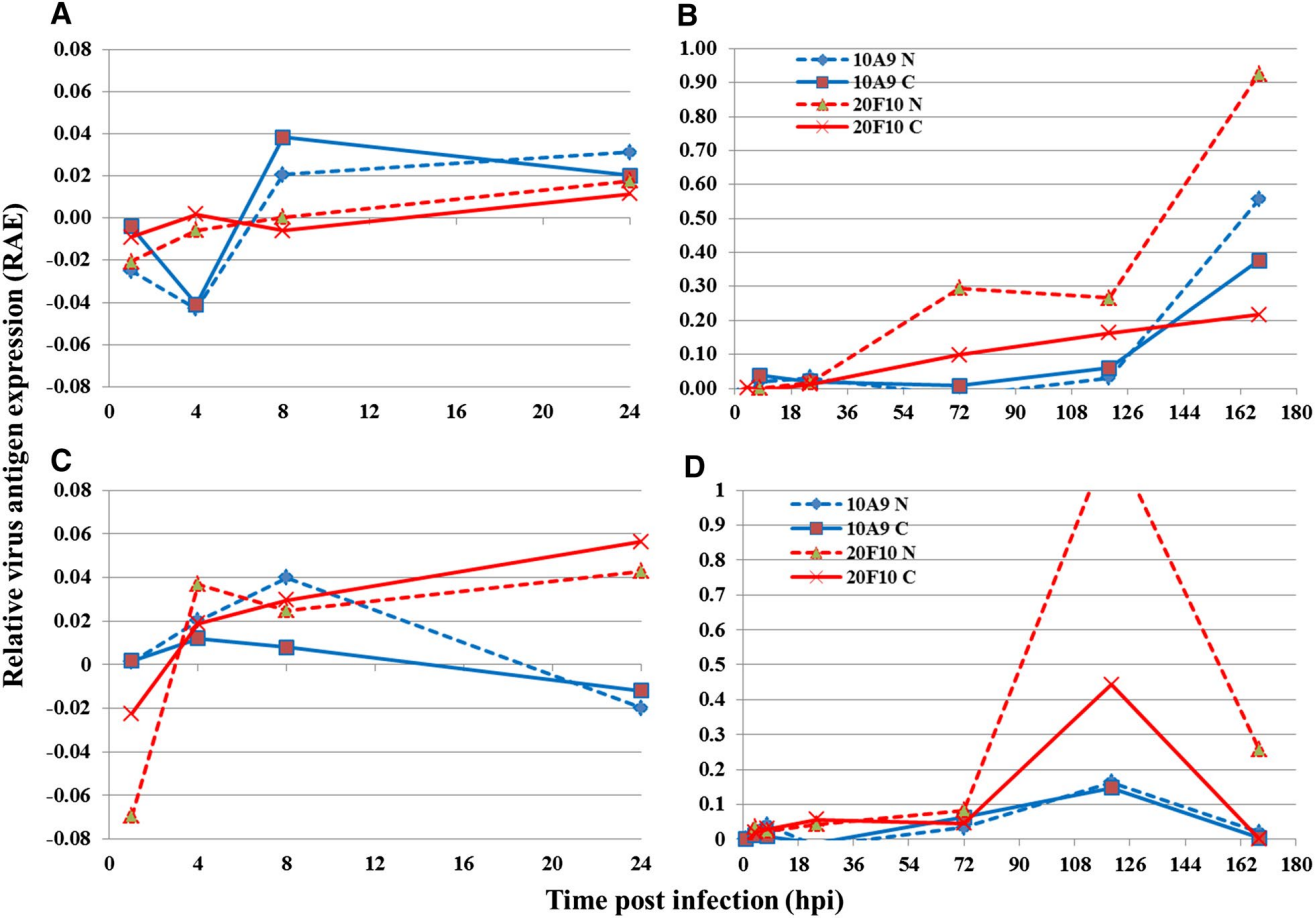
**CyHV-3 antigen expression using immunofluorescence of capsid-associated protein and envelope glycoprotein MAb on confocal micrographs by image analysis.** **A** CyHV-3 infected CCB cells first 24 hpi (n = 1 cell culture); **B** CyHV-3 infected CCB cells 1–7 dpi (n = 1 cell culture); **C** CyHV-3 infected KF-1 cells first 24 hpi (mean n = 2 cell cultures); **D** CyHV-3 infected KF-1 cells 1–7 dpi (mean n = 2 cell cultures). MAb 10A9 recognition of envelope glycoprotein, MAb 20F10 recognition of capsid associated protein.

It was difficult to observe the signals at the earliest stages on confocal micrographs alone, but utilising image analysis provided a tool in which to observe expression of the capsid-associated antigen as early as 4–8 hpi (Figure 4A, C), with an increase in expression noted after 24 hpi. After 3 dpi there was a dramatic increase in capsid-associated antigen expression, particularly associated with the nucleus, when >10-fold increased antigen expression was observed (Figure 4D), although not with both cell lines (Figure 4B). Expression of the capsid protein was much greater in the nucleus of the cell after 24 hpi compared to the cytoplasm (Figure 4B, D). Expression of the envelope glycoprotein was also detectable between 4 and 8 hpi (Figure 4A, C), although no substantial increase in its expression was noted until 5 dpi. Notably, the levels of glycoprotein antigen had surpassed those of the cytoplasmic capsid-associated antigen during later stages of the infection, but not the nuclear capsid-associated antigen (Figure 4B).

Although both cell lines generally facilitated similar levels of CyHV-3 antigen expression, differences were observed such that the expression of capsid-associated antigen was significantly greater during the later stages of infection (i.e. 2–7 dpi) compared to the earlier stages (i.e. 1–24 hpi) in CCB cells (*p* = 0.016), as was the expression of the envelope glycoprotein antigen (*p* = 0.05), but not in KF-1 cells. Interestingly, after 5 dpi there was a high abundance of envelope glycoprotein antigen within CCB cells, but levels fell in KF-1 cells, possibly due to many cells in this cell line having lysed by this stage (Figure 4B, D). Additional files 4 and 5 show the visible fluorescent signals during the time course after staining cells with the anti-CyHV-3 MAbs.

**Figure 5:**
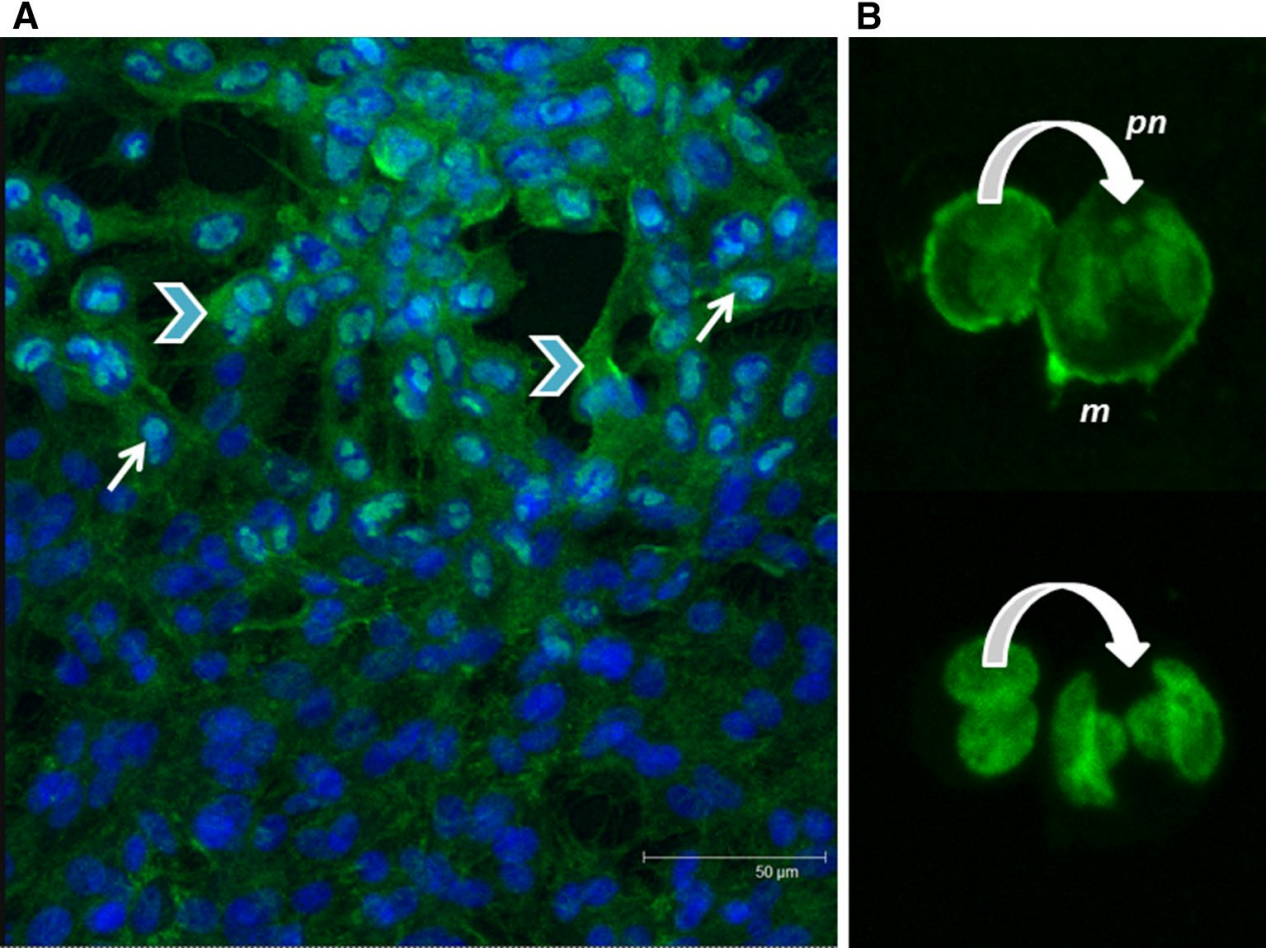
**Differential characteristics of CyHV-3 antigen expression observed by confocal microscopy in infected KF-1 cells.** **A** CyHV-3 infected cells labelled with MAb 20F10 detecting virus capsid-associated antigen. A confluent region of the KF-1 cell monolayer is shown with both cytoplasmic and concentrated nuclear signals of CyHV-3, 7 dpi; **B** CyHV-3 infected cells labelled with MAb 10A9 detecting virus envelope glycoprotein. Upper micrograph the signals seen in the infected cells shown are associated with the periphery of the nucleus (pn) and plasma membrane (m); Lower micrograph the nucleus of same infected cell as the upper micrograph is shown stained by propidium iodide—the curved arrow highlights the viral signal around periphery of nucleus. Blue DAPI/propidium iodide stained nuclei (in overlay micrographs) apart from the lower micrograph in **B**; green FITC staining of virus. Thin arrows nuclear associated antigen; curved arrows membranous associated antigen; arrow heads cytoplasmic associated antigen.

At later stages of infection, a high abundance of capsid-associated antigen was observed within both the nucleus and cytoplasm, particularly in highly confluent areas of the cell monolayer. In some cells, capsid-associated antigen signals were localised solely to the nucleus (Figure 5A). In contrast, the glycoprotein antigen was never expressed within the nucleus, although positive staining was often intense around the cell plasma membrane and occasionally associated with the periphery of the nucleus (Figure 5B).

### Immunogenicity of capsid-associated antigen

**Figure 6:**
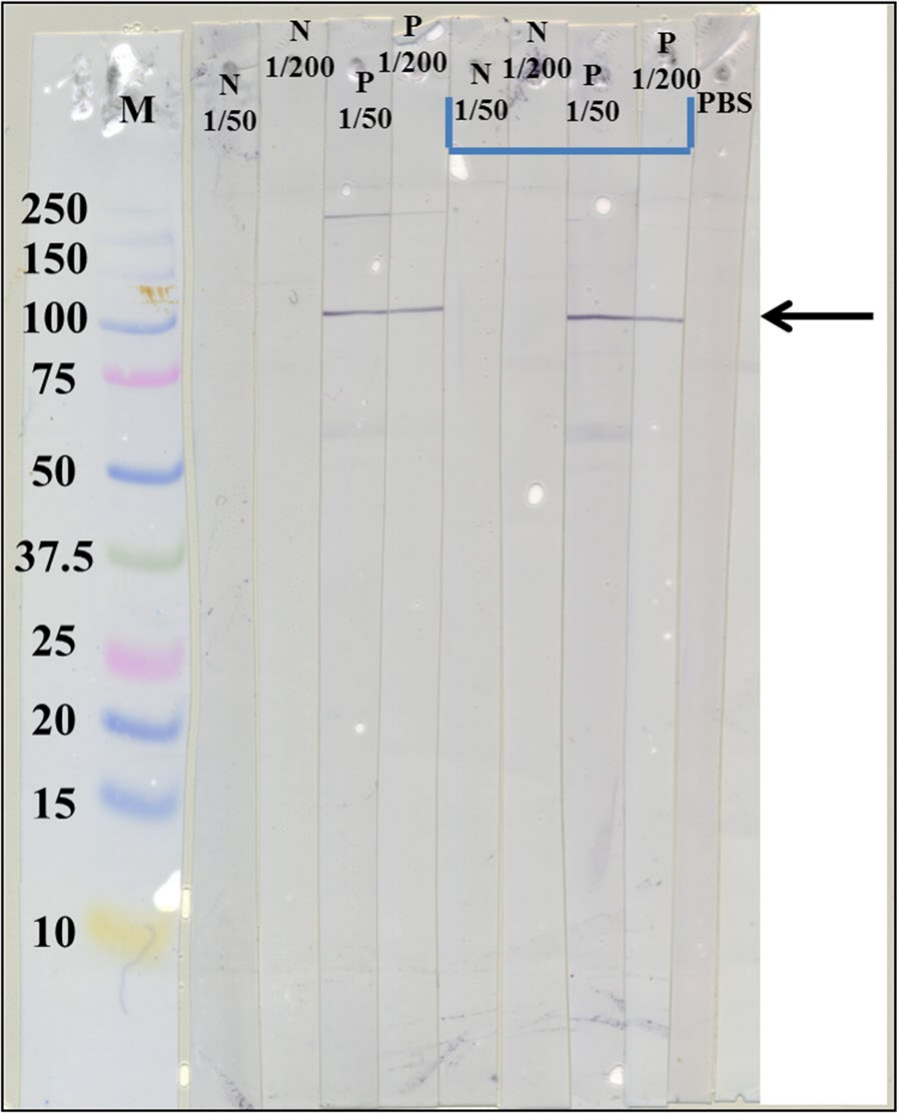
**Western blot screening of separated purified CyHV-3 polypeptides with carp anti-sera.** Strips of membrane with CyHV-3 polypeptides were incubated independently with carp sera as follows: N 1/50 negative sera diluted 1/50; N 1/200 negative sera diluted 1/200; P 1/50 positive sera diluted 1/50; P 1/200 positive sera diluted 1/200; PBS phosphate buffered saline control (no sera). Lane M molecular weight markers. Light blue underline indicates sera with diluent blocking (2% SMP). *Arrow* indicates strong immunoreactive band of ~100 kDa.

An intensely stained band of ~100 kDa was recognised by the sera sampled from the CyHV-3 infected koi by western blot (Figure 6). No bands were observed with sera sampled from naïve koi (Figure 6).

### Identification of 100 kDa capsid-associated antigen by MALDI-TOF/TOF MS

The 100 kDa protein band recognised by the infected carp sera was subsequently identified in MALDI-TOF/TOF analysis as an uncharacterised CyHV-3-specific protein encoded by ORF84 with an estimated molecular weight of 86 kDa according to the full CyHV-3 genomic sequence [35]. An additional figure shows the ~100 kDa band excised from the SDS-PAGE gel that was subsequently identified based on protein probability scores that were considered significant (*p* < 0.05) (see Additional file 6).

## Discussion

To date, although the proteome of CyHV-3 has been described in some detail [8, 10], information on the role and functional characteristics of the 156 proteins associated with the virus is limited. The use of molecular tools to identify the function of some of these proteins highlighted virulence factors, enzymes involved in nucleotide metabolism and proteins involved in immune-evasion [24, 36, 37], while immunofluorescence has been applied to examine immunogenicity and expression of the proteins [16]. Serological diagnostics for other viruses have been based on immunogenic viral antigens that are abundantly expressed during infection [38]. Thus, MAbs were utilised in the current study to assess the expression kinetics of potential diagnostically-relevant CyHV-3 antigens in vitro and these were then screened with sera from infected fish to determine their immunogenicity.

In the present study, 20–22 bands were observed after silver staining SDS-PAGE gels containing purified CyHV-3 virions. This is similar to the 21 bands found in Coomassie blue stained gels by Adkison et al. [39], but is less than the number of CyHV-3 bands observed in other studies, ranging between 25 and 31 polypeptides [8, 10, 22, 40]. Although variations between geographically-associated virus genotypes have been reported for CyHV-3 isolates [41, 42], homogeneity of the CyHV-3 proteome has been demonstrated by SDS-PAGE [40, 43]. The high proportion of CyHV-3 protein retained within the host cell, i.e. the high levels of capsid antigen still associated with the nucleus after 7 dpi, may also help to explain the loss of some virus proteins, following purification of virions on sucrose gradients by ultracentrifugation, as the antigens remain associated with cellular compartments. Variations in purified virus protein profiles of CyHV-3 have also been noted with and without the presence of glycosidases on different CyHV-3 isolates [8, 10].

The multiple bands observed in immunoblots were unexpected, as each MAb recognised more than one epitope on the blot. Previous characterisation of a MAb detecting the product of CyHV-3 ORF68 resulted in 3 bands, which were thought to be the result of ORF68 encoding a polyprotein and thus the three bands detected were either a result of cleavage by proteases, or alternative splicing or glycosylation of the protein after cleavage [18]. Both MAb 10A9 and 20F10 were used at 20 µg mL^−1^ and showed similar multiple banding by western blot. Fuchs et al. [16] observed similar molecular weight multiple banding on immunoblots screened with rabbit anti-sera raised to CyHV-3 ORF149—the target antigen of MAb 10A9. In herpesviruses, protease precursors undergo auto-processing and cleavage in order to achieve cleavage of subsequent polyproteins during capsid maturation and shell assembly [44], which can also lead to multiple bands being recognised by individual MAbs [45]. Alternatively, MAbs detecting glycoproteins may also recognise the antigen at multiple molecular weights due to a larger molecule being synthesised following glycosylation and other post-translational modifications resulting in the addition of oligosaccharides, and/or glycosylation intermediates [46, 47]. Protein processing and modifications during CyHV-3 infection may explain the bands observed, as the MAbs used here are known to detect a CyHV-3 capsid-associated antigen and envelope glycoprotein. An intensely stained lower molecular weight band at ~100 kDa, recognised only by MAb 20F10 could be due to it being a non-glycosylated capsid antigen. Large carbohydrate moieties associated with glycosylated proteins can inhibit electrophoretic migration through the gel by binding to SDS molecules [48]. This could therefore have influenced electrophoretic migration of the envelope glycoprotein antigens detected by MAb 10A9 but not the 100 kDa capsid associated protein antigen detected by MAb 20F10. This band was subsequently identified as a product of ORF84, a protein that Yi et al. [10] also identified by SDS-PAGE following deglycosylation, which supports the hypothesis above.

The semi-quantitative IFA performed in 96-well microtitre plates was developed for analysing CyHV-3 antigen expression during stages of virus replication and maturation, and followed a similar methodology reported for antigen quantitation by Xijier et al. [28]. Interestingly, elevated protein abundance was not detected by MAb 10A9, until the most advanced stages of virus infection, at which point the intensity of nuclear signal emitted by DAPI staining decreased, likely associated with lysis of infected cells at this late stage of infection. In contrast, progressively greater levels of protein were instead recognised by MAb 20F10 throughout the trial (up to ≥4-fold higher), which were significantly greater than that of the glycoprotein antigen between 2 and 7 dpi. Different cell lines used for CyHV-3 propagation have been reported to vary in their susceptibility to the virus [49], therefore two cell lines were used to validate the expression kinetics of the antigens examined in the current study. While both cell lines exhibited similar expression trends for the antigens, differences were observed and suggested that CCB cells retain greater levels of both enveloped and non-enveloped intracellular virions, while KF-1 cells are more prone to lysis releasing mature virions.

In order to validate the results obtained using the high throughput microtitre plate IF assay, a more sensitive confocal laser microscopy image analysis technique was developed enabling the detection of envelope glycoprotein antigen after just 4–8 hpi. This CyHV-3 envelope glycoprotein recognised by MAb 10A9 is a product of ORF149 [16] and transcripts of this ORF, characterised as an immediate early gene, have previously been detected as early as 2 hpi in CyHV-3 infected CCB cells, prior to DNA synthesis, which commences after 4–8 hpi [11]. Herpesvirus envelope glycoproteins, such as gB of HSV-1, are synthesised prior to DNA replication, albeit in small amounts [50], thus the early detection of this CyHV-3 glycoprotein antigen may be prior to DNA synthesis, glycosylation, and other post translational modifications. Furthermore, as this early expression is also associated with the nucleus (as quantified by image analysis), but not within the nucleus (as observed by confocal microscopy) (Figures 4B, D, 5B) the protein may be present in the nuclear envelope as well as being abundant in extracellular virions, similar to the herpesvirus glycoprotein gB, which is found in both primary and secondary enveloped virions [51, 52]. Previous observations by Fuchs et al. using another MAb recognising the ORF149 envelope glycoprotein [16] corroborated these findings, suggesting that the protein is synthesised at the endoplasmic reticulum followed by maturation in the Golgi network. Examination of the localisation of the envelope glycoprotein during later stages of the infection revealed compartmentalised signals at the periphery of the nucleus and around the plasma membrane, but not within the nucleus. This may be explained by the antigen being recognised before post-translational modification, i.e. synthesis at the endoplasmic reticulum (peripheral nuclear staining with MAb 10A9) prior to its translocation to the Golgi apparatus for glycosylation. The concurrent plasma membrane signals could represent the integration of viral glycoprotein into the viral envelope for later budding events of the infectious virions at the cell membrane. Similar distributions of the highly conserved and immuno-dominant gB in HSV-1 [53] have been demonstrated [51, 52]. This supports the earlier hypotheses for the multiple protein bands observed in the western blot as the MAb may recognise the same protein epitope prior to glycosylation, similar to findings of gE and gI expression in Feline herpesvirus [46].

The capsid-associated antigen was also first detected after 4 hpi by confocal microscopy and image analysis, in line with previous detection of transcripts encoding for ORF84 [11], with similar levels of cytoplasmic and nuclear associated protein as that of the envelope glycoprotein antigen. It was not until 24 hpi that there was a pronounced difference in the expression and abundance of the 2 antigens, where an 8–10 fold increase in capsid-associated protein expression during the first 5 dpi was observed compared to a ≤2-fold increase in envelope glycoprotein expression. As the same concentrations of MAbs were used for screening (20 μg mL^−1^) and intense signals were noted for each MAb during the late stages of infection, i.e. 10 dpi, differences in the affinity of the MAbs was not considered to influence this outcome. Furthermore, stronger signals were actually observed for MAb 10A9 compared to 20F10 using enzyme linked immunosorbant assays with purified CyHV-3 coated plates (unpublished data). The greater expression of the capsid-associated antigen may be related to the subsequent infection in neighbouring cells and the high proportion of non-infectious immature virions (i.e. with capsid protein) compared to mature infectious enveloped virions containing glycoproteins. This capsid-associated antigen, being a product of ORF84, is expressed as an early gene which mirrors the expression kinetics of the major capsid protein of ORF92 at the transcript level [12].

The antigen recognised by MAb 10A9 has been detected in peripheral blood leukocytes of fish thought to be a potential reservoir for the virus, such as sturgeon and goldfish [21, 54], suggesting that this antigen is not easily degraded in vivo. Furthermore, MAb 10A9 was recently used to affinity purify CyHV-3 viral proteins in asymptomatic-infected carp tissues that were negative by thymidine kinase (TK) PCR [55], which successfully yielded 5 CyHV-3 proteins and a large number of host proteins [23]. The membrane glycoproteins of CyHV-3 are good targets for vaccine development and sero-diagnostic test development, such as the abundant immunogenic products of the ORF25 gene family, i.e. ORF25, ORF65 and ORF149, which are detectable using KHV neutralising antibodies [16]. However, the highly expressed capsid-associated product of ~100 kDa, encoded by ORF84, has no known role in CyHV-3 assembly or antigenicity [8], but has recently been characterised as a marked CyHV-3 virion protein [10] and is recognised by experimentally CyHV-3 infected koi anti-sera, and thus may be a suitable candidate for development of a serological test for KHV. The variation in immunoreactivity of individual fish to different CyHV-3 antigens has been noted with different antigens in western blot analysis, however [17, 39]. Nonetheless, the antigen recognised in this study (~100 kDa) may be analogous to one of the more immunodominant proteins of CyHV-3 previously described, i.e. a 97 kDa protein that was recognised by 7/8 fish in the study by Adkison et al. [39]. This antigen was also previously found to be recognised by a number of experimentally infected fish with an Israeli isolate as well as CyHV-3 sero-positive fish from a surveillance programme conducted in Asia [56]. Importantly, this antigen does not appear to be conserved between other closely related alloherpesviruses, unlike another abundant immunogenic CyHV-3 capsid protein, the major capsid protein encoded by ORF92 [16, 35], thus potentially avoiding problems associated with cross-reactivity of antibodies to CyHV-1 that currently hampers CyHV-3 sero-diagnostic sensitivity and specificity [39, 56, 57]. Nonetheless, a larger number of CyHV-3 infected and naïve carp sera samples need to be tested in order to validate the usefulness of this antigen as a tool for KHV serodiagnostics.

In conclusion, greater capsid antigen expression and abundance was observed over envelope glycoprotein production using the novel semi-quantitative 96-well microtitre plate IF method and confocal microscopy-image analysis. The envelope glycoprotein expression, detected by MAb 10A9, was minimal until later stages of infection indicating a higher abundance of this protein is present on released whole virions, whereas a greater proportion of capsid antigen is produced and retained within the cell. Following cell lysis, the release of these abundant capsid antigens may expose them to carp B cells and thus they promote the production of specific antibodies against them. Abundant envelope glycoproteins, such as that of ORF149, that harbour neutralising epitopes are likely to play a role in virus attachment and penetration into the cell, and are thus good targets for vaccine development and diagnostic testing. However, determining highly abundantly expressed and immunogenic capsid antigens that induce non-neutralising antibodies, i.e. that expressed by ORF84, may also have particular potential in development of serological diagnostic tests for this notifiable pathogen.

## Additional files

10.1186/s13567-015-0297-4 **Graphical representation of fluorescence from MAbs 20F10 and 10A9 recognising Koi herpesvirus antigens over the course of infection in KF-1 cells in 96 well microtitre plates.** Graphs are of cells analysed during the first day of infection. (A) DAPI fluorescence of cells; (B) Difference of relative FITC to DAPI stain from blank wells at 0 hpi. Red line = MAb 20F10 (Capsid-associated antigen); Blue line = MAb 10A9 (Envelope glycoprotein antigen) Mean ± SE (*n* = 4 individual cell cultures).
10.1186/s13567-015-0297-4 **Graphical representation of fluorescence from MAbs 20F10 and 10A9 recognising Koi herpesvirus antigens over the course of infection in CCB cells in 96 well microtitre plates**. Graphs are of cells analysed during the first day of infection. (A) DAPI fluorescence of cells; (B) Difference of relative FITC to DAPI stain from blank wells at 0 hpi. Red line = MAb 20F10 (Capsid-associated antigen); Blue line = MAb 10A9 (Envelope glycoprotein antigen). Mean ± SE (*n* = 4 individual cell cultures).
10.1186/s13567-015-0297-4 **Transformation of confocal microscopy fluorescence for image analysis in CyHV-3 infected KF-1 and CCB cells screened with MAbs 10A9 and 20F10.** (A) Example for CyHV-3 infected CCB cells after 3 and 7 dpi screened with MAb 20F10. The parameter converted for image analysis is indicated above each column. Top row = confocal micrographs, bottom row = image utilised for image analysis subsequent to transformation (B) Images of quantification parameters utilised by the image analysis macro. The parameter measured for image analysis is above the micrographs and above this is the cell line used. Time post infection is indicated on the right of each row as well as the MAb used for screening. MAb 10A9 = Envelope glycoprotein MAb; MAb 20F10 = Capsid-associated MAb; ISA MAb = Irrelevant MAb detecting infectious salmon anaemia virus; PBS = phosphate buffered saline.
10.1186/s13567-015-0297-4 **Confocal micrographs of FITC fluorescence signals from CyHV-3 infected KF-1 cells screened with MAbs during infection.** The time of sampling is indicated left of the micrographs. The filter channels used during scanning the sections is indicated above the micrographs, i.e. the green channel only shows virus signals for glycoprotein (MAb 10A9) or capsid-associated (MAb 20F10) antigens and blue/green merge shows virus signal in relation to the cell nuclei (blue).
10.1186/s13567-015-0297-4 **Confocal micrographs of FITC fluorescence signals from CyHV-3 infected CCB cells screened with MAbs during infection.** The time of sampling is indicated left of the micrographs. The filter channels used during scanning the sections is indicated above the micrographs, i.e. the green channel only shows virus signals for glycoprotein (MAb 10A9) or capsid-associated (MAb 20F10) antigens and blue/green merge shows virus signal in relation to the cell nuclei (blue).
10.1186/s13567-015-0297-4 **1D SDS-PAGE gel of purified CyHV-3 polypeptides and identification by MALDI-TOF/TOF MS mass spectrometry.** (A) 1D SDS-PAGE of purified CyHV-3 polypeptides and excision of band ~100kDa. (B) Mascot search results of the 100kDa gel band excised from A. The red bar on the right (star) represents CyHV-3 ORF84 (CyHV-3 capsid-associated protein) and is significant. Protein scores greater than 84 are considered significant (*p* < 0.05).
