# Supplementary figures and images for: Expression of immunogenic structural proteins of cyprinid herpesvirus 3 in vitro assessed using immunofluorescence
Source: Vet Res. 2016 Jan 8;47:8. doi: 10.1186/s13567-015-0297-6 (PMC4705813; doi:10.1186/s13567-015-0297-6)

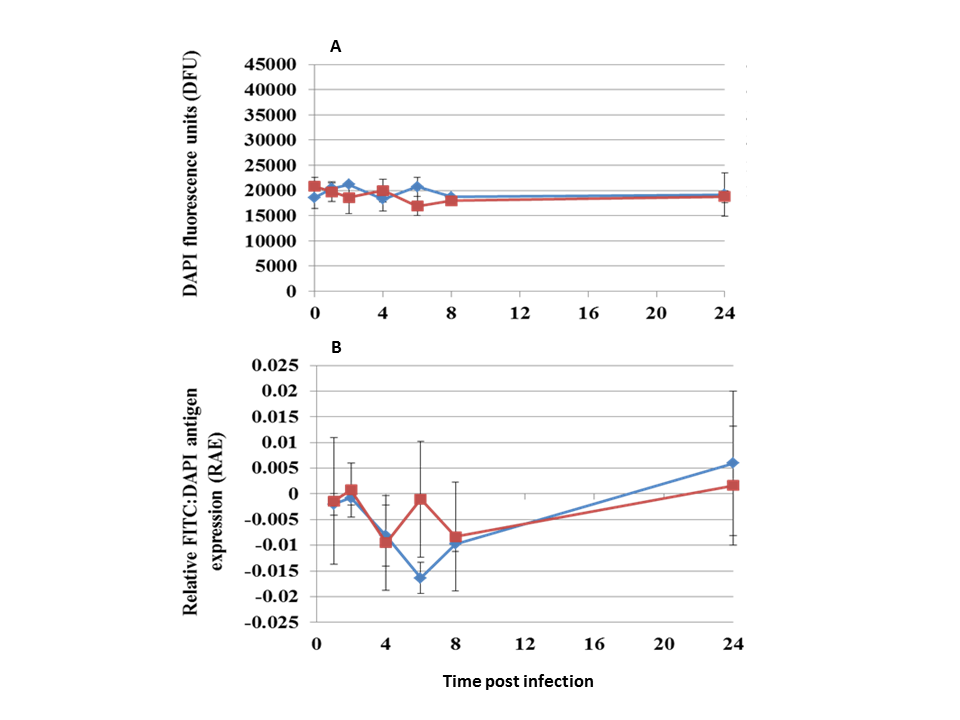

Supplement: Supplementary file 1 — 10.1186/s13567-015-0297-4 Graphical representation of fluorescence from MAbs 20F10 and 10A9 recognising Koi herpesvirus antigens over the course of infection in KF-1 cells in 96 well microtitre plates. Graphs are of cells analysed during the first day of infection. (A) DAPI fluorescence of cells; (B) Difference of relative FITC to DAPI stain from blank wells at 0 hpi. Red line = MAb 20F10 (Capsid-associated antigen); Blue line = MAb 10A9 (Envelope glycoprotein antigen) Mean ± SE (n = 4 individual cell cultures). [file 13567_2015_297_MOESM1_ESM.png]

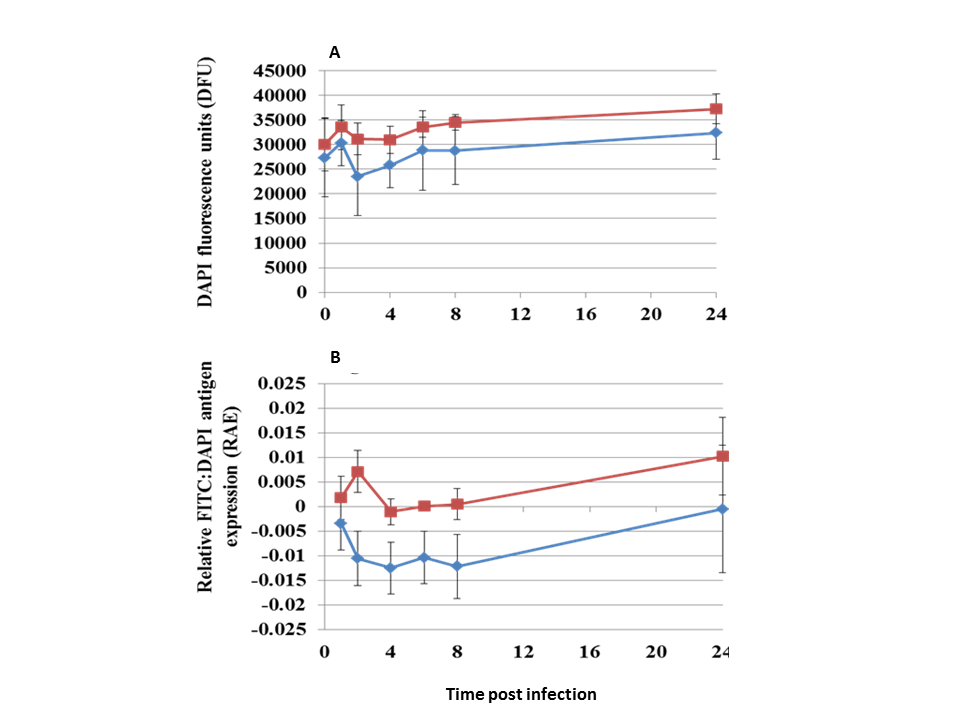

Supplement: Supplementary file 2 — 10.1186/s13567-015-0297-4 Graphical representation of fluorescence from MAbs 20F10 and 10A9 recognising Koi herpesvirus antigens over the course of infection in CCB cells in 96 well microtitre plates. Graphs are of cells analysed during the first day of infection. (A) DAPI fluorescence of cells; (B) Difference of relative FITC to DAPI stain from blank wells at 0 hpi. Red line = MAb 20F10 (Capsid-associated antigen); Blue line = MAb 10A9 (Envelope glycoprotein antigen). Mean ± SE (n = 4 individual cell cultures). [file 13567_2015_297_MOESM2_ESM.png]

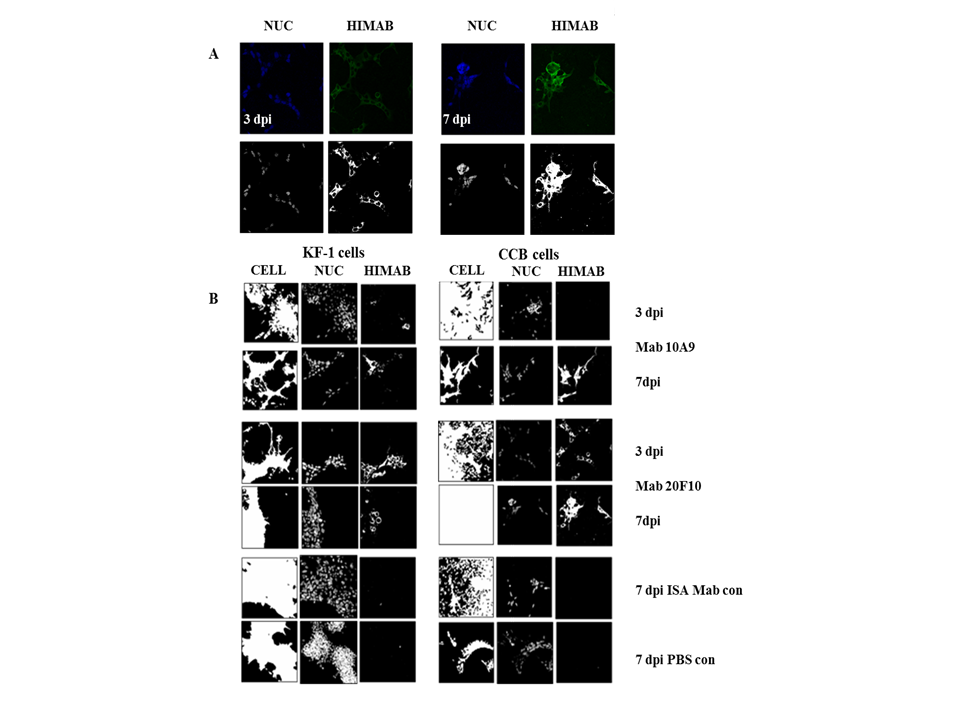

Supplement: Supplementary file 3 — 10.1186/s13567-015-0297-4 Transformation of confocal microscopy fluorescence for image analysis in CyHV-3 infected KF-1 and CCB cells screened with MAbs 10A9 and 20F10. (A) Example for CyHV-3 infected CCB cells after 3 and 7 dpi screened with MAb 20F10. The parameter converted for image analysis is indicated above each column. Top row = confocal micrographs, bottom row = image utilised for image analysis subsequent to transformation (B) Images of quantification parameters utilised by the image analysis macro. The parameter measured for image analysis is above the micrographs and above this is the cell line used. Time post infection is indicated on the right of each row as well as the MAb used for screening. MAb 10A9 = Envelope glycoprotein MAb; MAb 20F10 = Capsid-associated MAb; ISA MAb = Irrelevant MAb detecting infectious salmon anaemia virus; PBS = phosphate buffered saline. [file 13567_2015_297_MOESM3_ESM.png]

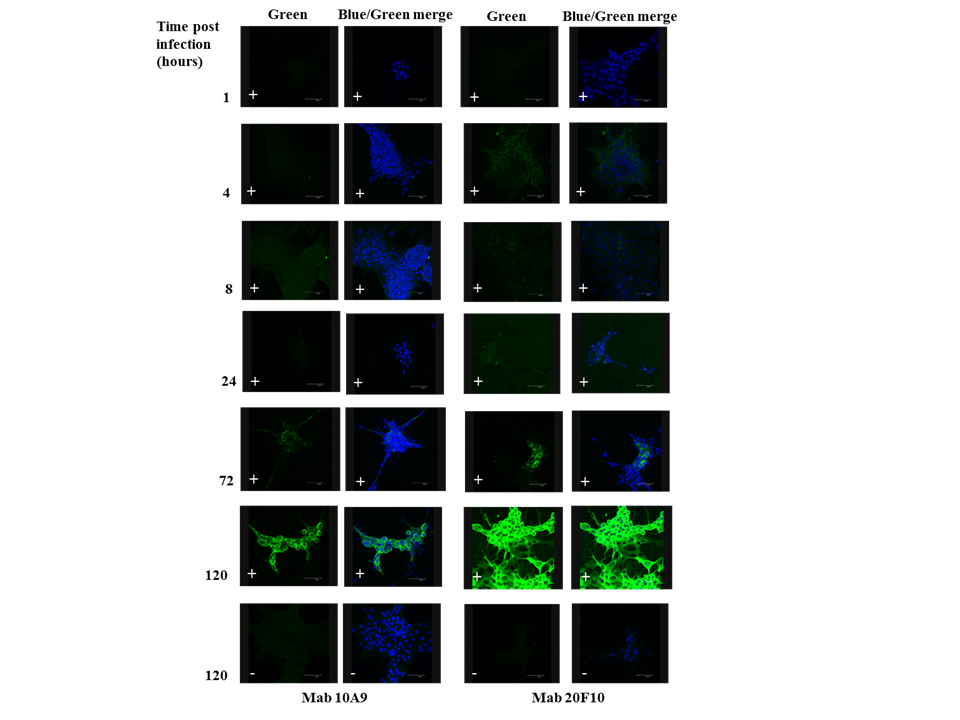

Supplement: Supplementary file 4 — 10.1186/s13567-015-0297-4 Confocal micrographs of FITC fluorescence signals from CyHV-3 infected KF-1 cells screened with MAbs during infection. The time of sampling is indicated left of the micrographs. The filter channels used during scanning the sections is indicated above the micrographs, i.e. the green channel only shows virus signals for glycoprotein (MAb 10A9) or capsid-associated (MAb 20F10) antigens and blue/green merge shows virus signal in relation to the cell nuclei (blue). [file 13567_2015_297_MOESM4_ESM.png]

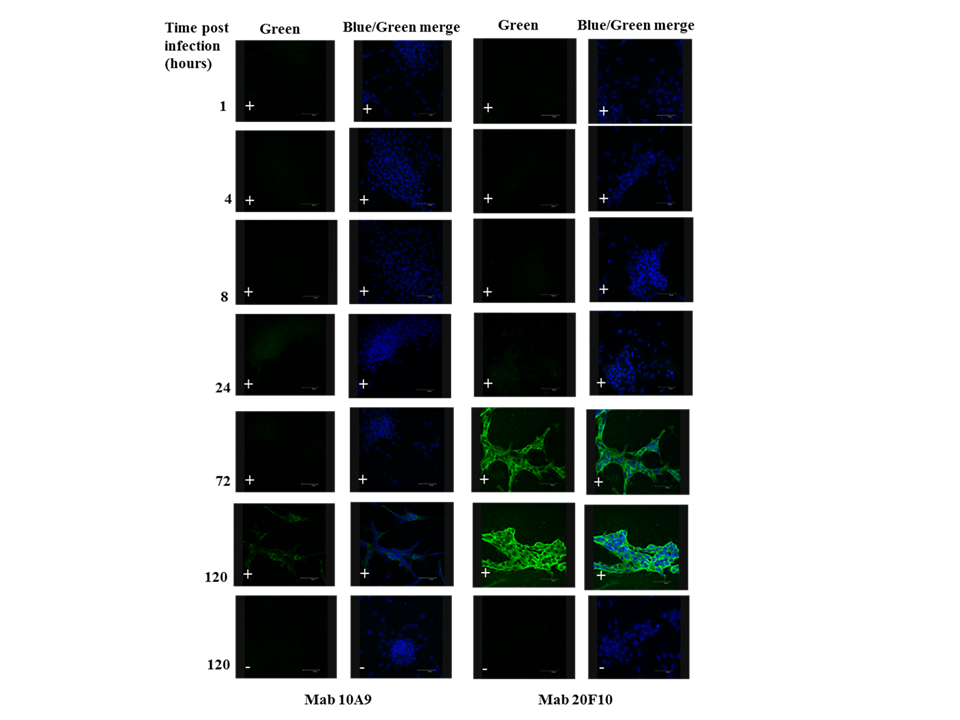

Supplement: Supplementary file 5 — 10.1186/s13567-015-0297-4 Confocal micrographs of FITC fluorescence signals from CyHV-3 infected CCB cells screened with MAbs during infection. The time of sampling is indicated left of the micrographs. The filter channels used during scanning the sections is indicated above the micrographs, i.e. the green channel only shows virus signals for glycoprotein (MAb 10A9) or capsid-associated (MAb 20F10) antigens and blue/green merge shows virus signal in relation to the cell nuclei (blue). [file 13567_2015_297_MOESM5_ESM.png]

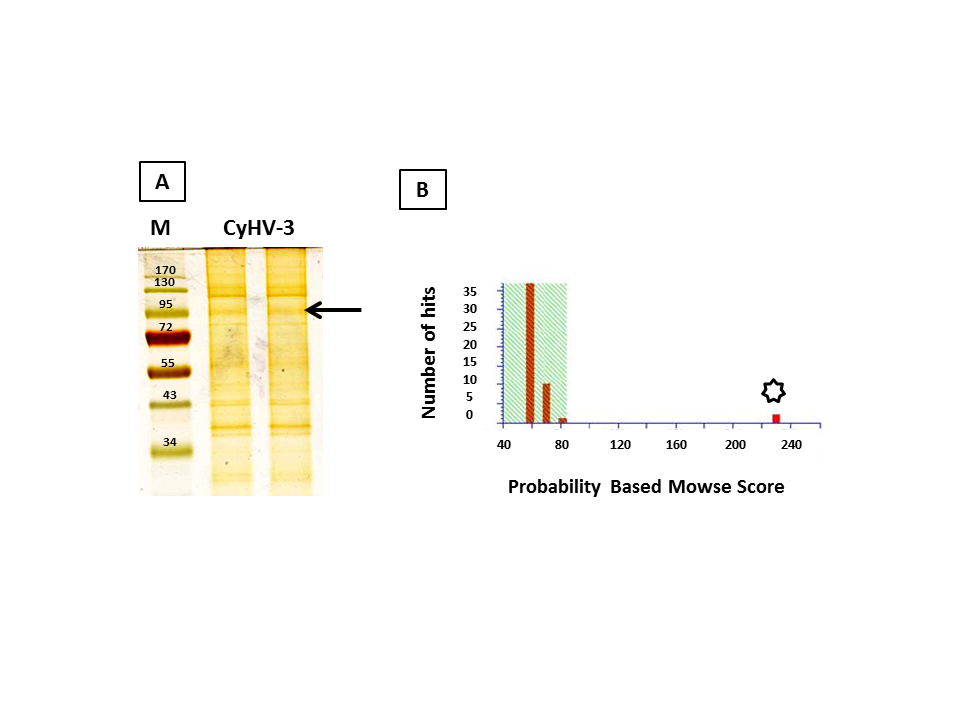

Supplement: Supplementary file 6 — 10.1186/s13567-015-0297-4 1D SDS-PAGE gel of purified CyHV-3 polypeptides and identification by MALDI-TOF/TOF MS mass spectrometry. (A) 1D SDS-PAGE of purified CyHV-3 polypeptides and excision of band ~100kDa. (B) Mascot search results of the 100kDa gel band excised from A. The red bar on the right (star) represents CyHV-3 ORF84 (CyHV-3 capsid-associated protein) and is significant. Protein scores greater than 84 are considered significant (p < 0.05). [file 13567_2015_297_MOESM6_ESM.png]
